# Qualitative and psychometric approaches to evaluate the PROMIS pain interference and sleep disturbance item banks for use in patients with rheumatoid arthritis

**DOI:** 10.1186/s41687-021-00318-w

**Published:** 2021-07-06

**Authors:** Brandon Becker, Kimberly Raymond, Carol Hawkes, April Mitchell Foster, Andrew Lovley, Cory Saucier, Avery A. Rizio, Jakob Bue Bjorner, Mark Kosinski

**Affiliations:** 1 GlaxoSmithKline, Upper Providence, PA USA; 2 Bristol Myers Squibb, Lawrenceville, NJ USA; 3 QualityMetric, Johnston, RI USA; 4 GlaxoSmithKline, London, UK; 5 QualityMetric, Copenhagen, Denmark

**Keywords:** Rheumatoid arthritis, Psychometric evaluation, Content validity, Concept elicitation, Cognitive debriefing, Patient-reported outcomes measurement information system, Patient-reported outcome, Pain, Sleep disturbance, Short form

## Abstract

**Background:**

Patients with rheumatoid arthritis (RA) commonly experience pain despite the availability of disease-modifying treatments. Sleep disturbances are frequently reported in RA, with pain often a contributing factor. The Patient-Reported Outcomes Measurement Information System (PROMIS) Pain Interference and Sleep Disturbance item banks were initially developed to provide insights into the patient experience of pain and sleep, respectively, though they were not specifically intended for use in RA populations. This study evaluated the content validity of the PROMIS Pain Interference and Sleep Disturbance item banks in RA and identified relevant content for short forms for patients with RA that achieved high measurement precision across a broad range of health.

**Methods:**

A qualitative approach consisting of hybrid concept elicitation and cognitive debriefing interviews was used to evaluate the content validity of the item banks in RA. Interviews were semi-structured and open-ended, allowing a range of concepts and responses to be captured. Findings from the qualitative interviews were used to select the most relevant items for the short forms, and psychometric evaluation, using existing item-response theory (IRT) item parameters, was used to evaluate the marginal reliability and measurement precision of the short forms across the range of the latent variables (i.e. pain interference and sleep disturbance).

**Results:**

Thirty-two participants were interviewed. Participants reported that RA-related pain and sleep disturbances have substantial impacts on their daily lives, particularly with physical functioning. The PROMIS Pain Interference and Sleep Disturbance item banks were easy to understand and mostly relevant to their RA experiences, and the 7-day recall period was deemed appropriate. Qualitative and IRT-based approaches identified short forms for Pain Interference (11 items) and Sleep Disturbance (7 items) that had high relevance and measurement precision, with good coverage of the concepts identified by participants during concept elicitation.

**Conclusion:**

Pain and sleep disturbances affect many aspects of daily life in patients with RA and should be considered when novel treatments are developed. This study supports the use of the PROMIS Pain Interference and Sleep Disturbance item banks in RA, and the short forms developed herein have the potential to be used in clinical studies of RA.

**Supplementary Information:**

The online version contains supplementary material available at 10.1186/s41687-021-00318-w.

## Introduction

Rheumatoid arthritis (RA) is a chronic autoimmune disease characterized by synovial inflammation, which results in damage to articular cartilage and underlying bone [1]. RA prevalence is greater in females than males and peaks in the 70–79 years of age group; in 2017 the estimated global prevalence was ~ 250 per 100,000 [2]. RA is associated with progressive disability, and increased disease severity is associated with negative impacts on health-related quality of life [3, 4]. Despite the availability of disease-modifying treatments, many patients with RA continue to experience pain; and > 10% of patients still experience significant levels of pain even when in remission (as measured by the disease activity score in 28 joints) [5]. Patients often identify pain as the symptom that they would most like to be improved [6].

Patients with RA often report that their sleep quality is impacted by the disease, and experience reduced sleep duration and daytime tiredness [7–11]. Studies have identified a complex association between pain and sleep in RA, with RA-related pain reported to be linked to sleep disturbances [8, 9, 11]. In one study, no significant correlation was found between overall disease activity and sleep quality, but a significant impact of pain severity on the duration of sleep was identified [8]. Similar associations between RA-related pain and sleep disturbance have been reported in several other studies [7, 9, 11, 12]; however, the directionality of this association is not clear [9, 13, 14]. For example, impacts to sleep have been shown to increase sensitivity to pain [15, 16], and poor sleep quality has been associated with increased pain severity in patients with RA and those with chronic pain [13, 14]. Another study observed a negative correlation between disease activity and daytime tiredness, which the authors suggested may be due to RA pain leading to increased alertness in the day [12]. These observations highlight the complex interplay between the different domains of health that are impacted by RA.

Studies investigating the symptoms of RA have traditionally measured pain in terms of severity, which is typically assessed in a clinical setting through the use of a visual analogue scale (VAS) or numeric rating scale (NRS) [17]. However, such measures of severity often provide only a one-dimensional insight into the manifestations of pain caused by RA [18, 19]. Instead, more complex and multi-faceted patient-reported outcome (PRO) measures have been developed to provide insights into the wide-ranging impacts of disease from a patient perspective [20]. Identifying appropriate outcome measures to assess the impact of pain on patients’ daily lives, as well as other meaningful endpoints such as sleep, is key to determining the benefits of a treatment [19]. However, there are several limitations associated with the use and interpretation of traditional PRO measures, including the lack of well-documented patient input into the development of instruments, a lack of sufficient measurement precision, and a greater likelihood of floor and ceiling effects [21–24].

The development of the Patient-Reported Outcomes Measurement Information System (PROMIS) helped to address several of the issues with traditional PROs [23, 25]. PROMIS is a set of PRO measures that encompass many areas of health and disease that, importantly, were calibrated in diverse population-based samples using item response theory (IRT). As a result, PROMIS item banks have the ability to be used flexibly across different populations and in various configurations, including short forms and computerized adaptive tests [24]. The PROMIS Pain Interference item bank v1.1 contains 40 items to assess a range of negative impacts from pain across seven subdomains: activities of daily living; cognition; emotional function; fun, recreation and leisure; sleep; social functioning; and sitting, walking, and standing [26, 27]. The PROMIS Sleep Disturbance item bank v1.0 consists of 27 items designed to evaluate perceptions of sleep quality, depth and restoration within the previous 7 days [28].

It can be more efficient to adapt an existing instrument where possible rather than developing a new PRO, provided that the content validity of the adapted instrument in the population of interest can be verified [29]. The PROMIS Pain Interference and Sleep Disturbance item banks were initially developed using clinical samples of patients with a variety of health conditions and large community-based samples that included healthy individuals and those with a range of health problems [26, 28]. Therefore, more focused research is required to support the relevance and understandability of these item banks in an RA population specifically, and to inform the selection of items for short forms that are most appropriate for patients with RA. Short-form versions of both the PROMIS Pain Interference and Sleep Disturbance item banks have been developed previously, which are more easily implemented in a clinical setting than the full item banks, but these were not tailored for use in an RA population [26–28].

In this study, we collected qualitative data to support the content validity of the PROMIS Pain Interference and Sleep Disturbance item banks in an RA population. Items that were identified as relevant for patients with RA from the item banks were considered for inclusion in short forms which could be used in clinical studies of RA. These items were further evaluated using the IRT item parameters established during the initial development of the item banks to ensure adequate coverage of the underlying concept across the range of the latent variable [26, 28]. Establishing content and psychometric validity of PRO measures for use in medical product development is consistent with recommendations from the Food and Drug Administration Guidance for Industry [30] and the International Society for Pharmacoeconomics and Outcomes Research (ISPOR) Clinical Outcome Assessment Emerging Good Practices Task Force [31].

## Methods

### Study design

**Fig. 1:**
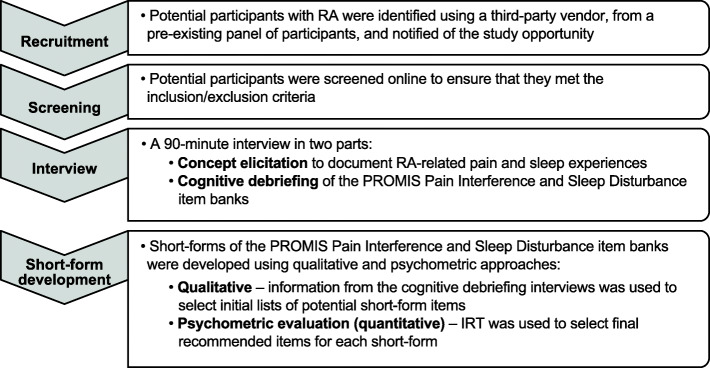
Study design. IRT, item response theory; PROMIS, Patient-Reported Outcomes Measurement Information System; RA, rheumatoid arthritis

A hybrid qualitative approach that employed concept elicitation and cognitive debriefing techniques was used to evaluate the content validity of the PROMIS Pain Interference and Sleep Disturbance item banks for use in RA (Fig. 1). The 90-min, one-on-one, audio-recorded interviews were conducted by experienced qualitative researchers trained on the specific objectives of the study. Qualitative interviews were conducted mostly in person to capture nonverbal and behavioral nuances important for interpreting cognitive debriefing interviews. A small number of interviews (*n* = 5) were conducted by phone to include participants who experience the most severe symptoms. Items for inclusion in the short forms of the PROs were subsequently identified using a mixed-methods approach consisting of qualitative analysis and quantitative psychometric evaluation. Qualitative data from the cognitive debriefing component were used to select initial candidate items for the short forms. Subsequent quantitative evaluation using established IRT item parameters was used to identify final recommended short forms. All study materials were approved by the New England Independent Review Board; tracking number 120180323.

### Study population and recruitment

Eligible participants had a self-reported clinician diagnosis of moderate/severe RA (and received diagnosis at ≥18 years); had been diagnosed with RA ≥2 years; had been in treatment for RA for the past 2 years; experienced symptoms of RA (e.g. joint pain/swelling) in the previous 7 days; reported ≥6 swollen joints and ≥ 6 tender joints at time of screening; and were fluent in English. Participants were excluded from the study if they were unwilling or unable to participate in an interview for 90 min to discuss their experience with pain related to their RA or had not received a conventional synthetic disease-modifying anti-rheumatic drug (csDMARD) and/or biologic treatment.

While there is no set number of interviews that can be specified a priori to confirm comprehensibility and relevance of a patient-reported instrument for hybrid interviews, the study sample size was based on ISPOR guidelines regarding the number of interviews needed to reach concept saturation and to establish how well participants understood the item content of each PROMIS item bank [31]. As each interview was limited to 90 min to reduce study participant fatigue, each participant was interviewed on only one item bank. This required recruitment of a greater number of participants in order to reach concept saturation for both item banks. More participants were asked to debrief the Pain Interference item bank than the Sleep Disturbance item bank due to the greater number of items in the former.

Prior to the qualitative interviews, a third-party recruitment vendor identified, screened, and scheduled participants located in the United States. All participants signed the informed consent form prior to attending the in-person qualitative interview. Participants were asked to provide their prescribed RA medications to the in-person interview, which were viewed and recorded by the interviews, as a means of indirectly confirming a physician diagnosis of RA. Approximately half of participants who participated in person (*n* = 15/27; 47%) brought their RA medication.

### Concept elicitation and cognitive debriefing

Qualitative interviews were conducted by experienced interviewers using a semi-structured interview guide with open-ended questions. The concept elicitation segment gathered spontaneously elicited descriptions from participants of their experience with RA-related pain and sleep disturbances. Targeted probes were developed in advance of the interviews, to clarify and further explore participant experiences. Interviewers were also trained to probe for clarification of responses when needed. Example questions from the concept elicitation component include *“How does pain from RA impact your life and how well you are able to function?”,* and *“Please tell me how RA has impacted your ability to fall asleep”*.

Following concept elicitation, cognitive debriefing interviews of either the PROMIS Pain Interference or the PROMIS Sleep Disturbance item bank were conducted using a think-aloud method [32], which encourages participants to verbalize their thought processes while choosing a response to the item stem. This method of interviewing assesses whether participants understand the item as intended by the developer and highlights any areas of difficulty related to the item. Participants were asked to note any aspects that they found confusing, conceptually redundant, or not relevant to their experiences with RA. Participants were asked to comment on the comprehensiveness of the items and whether there were any relevant parts of their disease experience not covered by any of the items. Participants also answered structured questions related to the instructions, response options, recall period and items included in the PROMIS item banks. Participant responses concerning each item were evaluated during coding and analysis for comprehension, clarity, and relevance to the participant’s experience of RA.

### Qualitative data coding and analysis

Anonymized transcripts of audio recordings were content coded and verified by trained QualityMetric qualitative researchers and reviewed by the qualitative primary investigator. Consensus meetings were held regularly with the research team, and a portion (*n* = 12; 38%) of the transcripts were double coded to target accuracy and reliability between coders. Data were coded and analyzed using NVivo version 11.0 (QSR International Pty Ltd.; Chatstone, Victoria, Australia).

Coding and analysis for the concept elicitation segment of the interviews was carried out according to grounded theory analysis [33], whereby concepts were allowed to emerge from participants rather than being imposed a priori*.* The item content of the PROMIS banks was then mapped back to information that participants elicited freely*.* Concept saturation, defined as the point at which no new concepts emerged from the interviews, was analyzed to confirm that enough interviews were completed to fully understand concepts important to patients related to pain interference and sleep disturbance [33]. Transcripts were coded in 4 sets of 8 transcripts by 2 qualitative researchers, using an iterative process. The first set of transcripts was coded to obtain an initial conceptualization of the data and identify major themes that were common across participants. The coders and the qualitative principal investigator met after coding the first set to discuss any discrepancies and to establish a set of codes to be used for subsequent interviews. The first set of transcripts was then re-coded using the agreed set of codes. Any changes to the set of codes following the first set of interviews were discussed and agreed upon by the coders and qualitative principal investigator.

Narrative data for the cognitive debriefing segment of the interviews were coded using a series of summarized ratings for content related to elements of each item bank (e.g. instructions, response choices, recall period, and each item) to determine whether participants found each element to be comprehensive, relevant and understandable. Each item was assigned 3 codes: one for whether a problem was reported for the item, a second for whether the item was reported as not relevant, and a third for whether this was a spontaneous or prompted remark. Each item was also assigned a code to indicate whether participants described the item as conceptually redundant, and which of the grouped redundant items were most preferred by the participant.

### Item selection for short forms

The full PROMIS Pain Interference and Sleep Disturbance item banks provide a source from which short forms can be adapted and, as such, some items may be considered to have conceptual redundancy with others or may not be relevant to a particular health condition. The development of a short form provides a refined selection of items deemed to be most relevant to a specific population. Selection of items for the short forms for use in RA populations was performed in two stages. First, to initially select potential items for inclusion in the short forms, items that were reported to be irrelevant or problematic by ≥25% of participants were recommended for removal. This threshold was set a priori*.* Second, to reduce the size of the item pool further, the remaining items were assessed and items that ≥25% of participants reported to be redundant with other items were collected into subsets. In each subset, the item that participants most often preferred was selected.

Following this selection of candidate items using the qualitative evidence, psychometric evaluation was performed (described in ‘Statistical analysis’) to develop short forms with high measurement precision across the range of pain interference or sleep disturbance experiences reported by patients with RA [34]. Psychometric evaluation assisted in the selection of items for the short forms by further reducing the degree to which redundant items appeared, and to guide the choice of items if patient preference was not clear during the interviews.

### Statistical analysis

For psychometric evaluation, published IRT parameters were obtained for the Pain Interference item bank v1.1 [26, 27] and the Sleep Disturbance item bank v1.0 [28]. Test information function and standard error of measurement were calculated from IRT model parameters and plotted for different combinations of short forms; the latent variable (Pain Interference or Sleep Disturbance) was plotted on the x-axis, and either test information, standard error of measurement, or marginal reliability was plotted on the y-axis. This allowed for marginal reliability comparisons across different short form item combinations, and with the original item bank. The marginal reliability of item sets was calculated to evaluate the impact of certain items on the overall item bank reliability, with a reliability score ≥ 0.90 indicating a scale with the precision to detect differences or changes in scores at the individual participant level with a high degree of certainty [35]. IRT simulation studies using 10,000 simulated responses were used to estimate floor and ceiling effects for RA samples with typical levels of pain interference and sleep disturbance [34], in order to determine whether adding or removing an additional item would have a desirable impact on the properties of the short forms. Expected a posteriori scoring was used for estimation of the highest and lowest possible IRT scores of item banks. All scores are reported in the standard normal metric with a mean of 0 and a standard deviation of 1 in the general population. For both domains, higher scores imply a worse impact upon health (i.e. more pain interference or sleep disturbance).

## Results

### Study population

A total of 32 participants were interviewed (PROMIS Pain Interference *n* = 20/32; PROMIS Sleep Disturbance *n* = 12/32). Participants were predominantly female (*n* = 21/32; 66%) and white (*n* = 20/32; 63%) (Table 1). The mean age was 53.9 years and the mean time since RA diagnosis was 10.7 years. Most patients (*n* = 26; 81%) did not know the stage of their RA.

**Table 1:** Participant demographics

	Total sample (***N*** = 32)	Pain interference (***n*** = 20^**a**^)	Sleep disturbance (***n*** = 12^**a**^)
Age (years), mean (SD)	53.9 (10.3)	53.2 (11.4)	55.0 (8.5)
Age at diagnosis (years), mean (SD)	42.6 (9.5)	42.4 (10.5)	42.9 (8.1)
Time since diagnosis (years), mean (SD)	10.7 (9.4)	9.2 (7.4)	13.3 (12.0)
Number of swollen joints at screening, mean (SD)	9.4 (4.4)	8.5 (4.0)	10.9 (4.8)
Number of tender joints at screening, mean (SD)	10.2 (6.2)	8.8 (3.6)	12.7 (8.8)
Female, n (%)	21 (66)	13 (65)	8 (67)
Race/ethnicity, n (%)			
White, non-Hispanic	20 (63)	14 (70)	6 (50)
Black or African American	10 (31)	4 (20)	6 (50)
Asian	1 (3)	1 (5)	0
Other	1 (3)	1 (5)	0
Current RA medication, n (%)			
Biologic only	1 (3)	1 (5)	0
csDMARD only	13 (41)	6 (30)	7 (58)
Both biologic and csDMARD	17 (53)	12 (60)	5 (42)
Neither biologic nor csDMARD^b^	1 (3)	1 (5)	0
Self-reported RA severity, n (%)			
Mild	0	0	0
Moderate	22 (69)	14 (70)	8 (67)
Severe	10 (31)	6 (30)	4 (33)

*csDMARD*, conventional synthetic disease-modifying anti-rheumatic drug; *RA*, rheumatoid arthritis; *SD*, standard deviation

^a^n values represent the number of participants who took part in cognitive debriefing for each item bank

^b^One participant had previously taken a biologic for treatment of RA, but was in the process of switching medications due to reported medication side effects

### Concept elicitation

#### Saturation analysis

A total of 50 concepts emerged from the interviews across seven major themes selected for saturation evaluation: physical functioning, emotional functioning, role functioning, social functioning, activities of daily living, cognitive functioning, and sleep disturbance (Tables 2 and 3). In the saturation analysis, 94% of the concepts were identified in the first 16 interviews, indicating that 32 interviews were sufficient to reach saturation.

**Table 2:** Impact of pain interference in patients with RA

Areas of impact due to pain, and representative quote	Number of participants reporting impact (***N*** = 32)
**Physical functioning**	**32**
*“I can’t go out and do a nice long walk with my husband. I can go halfway up the block and back, that’s about it.” (Female, age 66)* *“I won’t be able to write – sometimes, … with the inflammation, is really bad, it’s a struggle.” (Female, age 38)* *“I don’t really do that many recreational activities anymore. I just can’t really do them, except activities where I just sit there and watch things, but then even that get a little bit difficult because it gets uncomfortable to sit in one position.” (Female, age 48)*	
Lower body impairments	32
*Standing*	*30*
*Walking*	*25*
*Sitting*	*20*
*Kneeling, crouching, bending*	*15*
*Staying still*	*14*
*Climbing stairs*	*11*
*Running*	*10*
*Pressing down*	*1*
*Lifting with legs*	*1*
Upper body impairments	28
*Grip strength, manual dexterity*	*26*
*Lifting with arms/hands*	*12*
*Raising arms*	*7*
*Staying still*	*2*
Impact to leisure, recreational or exercise	24
*Fatigue, lethargy*	*9*
Difficulty adjusting the body due to pain from RA	*4*
*Sexual dysfunction*	*2*
**Emotional functioning**	**32**
*“I was just thinking how much [pain from RA has] affected my life and how miserable I am.” (Female, age 52)*	
*Sadness, depression*	*24*
*Irritability, snappishness*	*17*
*Difficulty enjoying life/activities*	*15*
*Discouraged*	*14*
*Frustration, stress*	*14*
*Fear, anxiety*	*10*
*Burdensomeness*	*8*
*Anger*	*5*
*Embarrassment*	*5*
*Self-consciousness*	*5*
*Increased sensitivity*	*3*
*Guilt*	*2*
**Role functioning**	**32**
*“[Pain from RA] was affecting my other job because my other job required me to move around more. I had to get a job now where I can sit down in front of two monitors.” (Male, age 45)*	
*Difficulty performing chores/errands*	*29*
*Impacts on work*	*21*
*Role changes, needing assistance*	*19*
*Difficulty leaving the house*	*13*
*Difficulty driving*	*4*
**Social functioning**	**31**
*“I don’t go out, … because of the pain. I don’t like being around a bunch of people.” (Male, age 65)*	
*Unable to participate*	*29*
*Personal relationships*	*22*
*Social isolation*	*19*
*Difficulty planning, having to cancel*	*16*
*Misunderstood by others*	*15*
*Masking symptoms*	*6*
**Activities of daily living**	**23**
*“Some days I think, “Oh, I’m going to take a long walk.” I don’t – that day I might not even get out of bed. That day I might not even be able to put my shoes on. Because my feet hurt so bad.” (Female, age 36)*	
*Reduced activity*	*17*
*Difficulty dressing*	*13*
*Difficulty washing/grooming*	*10*
*Difficulty toileting*	*5*
*Difficulty eating*	*1*
**Cognitive functioning**	**22**
*“If I’m concentrating on a task at hand and I’m having to do some things, [the pain] very much hampers my concentration because I cannot actually fulfill what I’m trying to do because I’m too busy paying attention to my pain.” (Male, age 37)*	
*Difficulty concentrating*	*19*
*Difficulty remembering*	*9*

**Table 3:** Impact of sleep disturbance in patients with RA

Type of sleep disturbance	Number of participants reporting (***N*** = 31)	Representative quote
Difficulty finding a comfortable position	28	*“It’s just that you have to take a lot of time, um, trying to do what’s comfortable for you, to even get an hour’s worth of sleep. So a lot of people use the wedge pillows for their knees, and things like that” (Female, age 51)*
Difficulty staying asleep	26	*“I get up every 2 hours, or 3 hours with the pain. Pop a pill, go back to bed.” (Female, age 61)*
Difficulty falling asleep	21	*“It’s the pain in my hands, that throbbing kind of pain that makes it difficult to go to sleep, but it doesn’t keep me from sleeping. It’s just the falling asleep part.” (Male, age 67)*

#### Pain interference

Participants indicated that pain from RA has substantial impacts on their daily lives, particularly related to physical, social, role, and emotional functioning (Table 2). In terms of physical functioning, almost all participants described experiencing impairments with both their lower body (*n* = 32/32) and upper body (*n* = 28/32). Pain from RA was reported to impact leisure, recreational, or exercise activities in the majority of participants (*n* = 24/32). All participants described how pain from RA affected their ability to carry out various roles and responsibilities, such as chores and errands, and most participants reported feeling sadness or depression (*n* = 24/32) due to the inability to lead independent and fulfilling lives. Furthermore, impacts in one area of a participant’s daily life often directly contributed to another; for example, many participants reported that physical impairments related to their RA pain restricted their ability to participate in social activities.

#### Sleep disturbance

Thirty-one participants were queried about their sleep habits, of which all (*n* = 31/31) reported difficulties with sleep due to RA-related pain. Common forms of disturbance included difficulty finding a comfortable position (*n* = 28/31), difficulty staying asleep (*n* = 26/31), and difficulty falling asleep (*n* = 21/31) (Table 3). Participants described how constant shifting is often required to find an adequate sleeping position, and that inadvertent movements triggered pain that caused them to awaken. Participants described consistently experiencing difficulties in falling or staying asleep. Participants reported that sleep disruptions due to RA contributed to a feeling that they did not get adequate rest (*n* = 24/31); most commonly, this impact was experienced as fatigue/lethargy during the day (*n* = 17/31), which often hampered productivity.

### Cognitive debriefing

#### Pain interference item bank

During cognitive debriefing of the PROMIS Pain Interference item bank, all participants (*n* = 20/20) reported that the instructions and response options were clear and easy to understand. Most participants (*n* = 19/20) reported that a recall period of 7 days was appropriate; one participant preferred a daily diary. Half (*n* = 10/20) suggested that longer recall periods of 2 weeks or 1 month could be used to better capture the variability in pain severity and interference if the instrument were not administered regularly to capture such variation.

Overall, participants reported that most items were relevant to their experience with RA. When asked to identify the most relevant items, participants generally identified subdomains of items as being the most relevant (e.g. “those that ask about social activities”), rather than identifying specific items. The most relevant groups of items were identified as standing, sitting, and walking (*n* = 10/20), and work or work around the home/household chores, errands or trips from home (*n* = 9/20). Participants reported that several items overlapped within each subdomain of the item bank (excluding the 1-item sleep subdomain within the Pain Interference item bank). Four items were considered not relevant or problematic by ≥25% (*n* ≥ 5) of participants (Table 4). The remaining items were assessed, and those deemed conceptually redundant by ≥25% of participants were collected into subsets from which participants could select their most preferred item. Following this step, a further 9 items were removed (Supplementary Table 1). Missing concepts reported by participants were related to symptoms (*n* = 7; most commonly fatigue [*n* = 5]), pain characteristics (*n* = 9; most commonly bodily location and severity [both *n* = 4]), and other interferences (*n* = 11; most commonly sleep [*n* = 7]).

**Table 4:** Items reported to be not relevant or problematic by ≥25% of participants interviewed for the Pain Interference or Sleep Disturbance item banks

Item	Not relevant, n	Reported problems, n	Reported problems or not relevant, n
**Pain interference item bank (*N*** **= 20)**			
PAININ1: How difficult was it for you to take in new information because of pain?	7	3	8
PAININ49: How much did pain interfere with your ability to remember things?	7	0	7
PAININ51: How often did pain prevent you from sitting for more than 10 min?	7	0	7
PAININ11: How often did you feel emotionally tense because of your pain?	4	2	5
**Sleep disturbance item bank (*N*** **= 12)**			
SLEEP65: I felt physically tense at bedtime	7	6	9
SLEEP70: I felt sad at bedtime	7	1	8
SLEEP72: I tried hard to get to sleep	4	3	6
SLEEP67: I worried about not being able to fall asleep	5	0	5
SLEEP68: I felt worried at bedtime	4	0	4
SLEEP93: I was afraid I would not get back to sleep after waking up	4	0	4
SLEEP20: I had a problem with my sleep	1	3	4
SLEEP45: I laid in bed for hours waiting to fall asleep	3	0	3
SLEEP116: My sleep was refreshing	1	2	3
SLEEP125: I felt lousy when I woke up	1	2	3

Participants could report that an item was problematic and not relevant; columns are not mutually exclusive and values represent the number of unique participants reporting problematic and/or relevant items. PROMIS items© 2008–2021; reprinted with permission, David Cella

#### Sleep disturbance item bank

During the cognitive debriefing interviews of the PROMIS Sleep Disturbance item bank, all participants were able to answer the items using the response options provided about their sleep disturbances related to RA. Most participants (*n* = 9/12) reported that the items were clear, easy to understand and answer, and captured important concepts related to sleep disturbance from RA. Those who reported some concern regarding answering items accurately (*n* = 3/12) were currently taking sleep medication to manage sleep disturbances and reported that their responses on the sleep disturbance scale may be slightly underestimated on the evenings when they take sleep medication.

Eleven of the 12 participants reported that the response options were easy to understand. Seven participants were explicitly asked to provide feedback on the instructions; all reported they were clear, simple, and easy to understand; the remaining five did not spontaneously report difficulty with the instructions. All 12 participants reported that recalling over a 7-day period was appropriate. Two participants suggested longer recall periods (e.g. since diagnosis, past 2 weeks) to capture the variability or fluctuations of RA and sleep disturbances if the measure was not administered at repeated and regular intervals.

The items most often reported as relevant to RA and sleep experience were ‘*I had trouble getting into a comfortable position to sleep’* (*n* = 4/12), and ‘*I had difficulty falling asleep’* (*n* = 3/12). Ten items were considered not relevant or problematic by ≥25% (*n* ≥ 3) participants (Table 4). Following the selection, by participants, of one preferred item from subsets of conceptually redundant items, an additional 6 items were removed (Supplementary Table 2). Participants reported missing concepts contributing to sleep disturbance that included both factors not directly related to RA (*n* = 4; such as sleep hygiene and temperature), as well as factors that were related to RA (*n* = 3; such as treatment or management of pain before sleep, and the specific RA symptoms that impacted sleep).

### Item selection for short forms for pain interference and sleep disturbance

Based on the cognitive debriefing findings, 27 Pain Interference items and 11 Sleep Disturbance items were considered by participants to be clear, unproblematic and relevant.

#### Pain interference short form

Due to the large number of potentially relevant Pain Interference items, concept elicitation data and psychometric evaluation were used to further guide reduction to 13 items that were not redundant or overlapping and that adequately covered all Pain Interference subdomains (activities of daily living; cognition; emotional function; fun, recreation and leisure; sleep; social functioning; and sitting, walking, and standing) [26], aside from sleep impacts. This 13-item Pain Interference bank was found to provide adequate precision for measuring the latent variable compared with the test information function using the full 40-item bank and the 27 items derived from the qualitative analysis. Measurement range was not overly compromised with the 13-item scale (IRT score range: − 1.07 to 3.09) compared with the initial 27-item scale (IRT score range: − 1.28 to 3.43), particularly in the range of scores that patients with active RA were likely to occupy (> 0 on the latent variable scale).

**Fig. 2:**
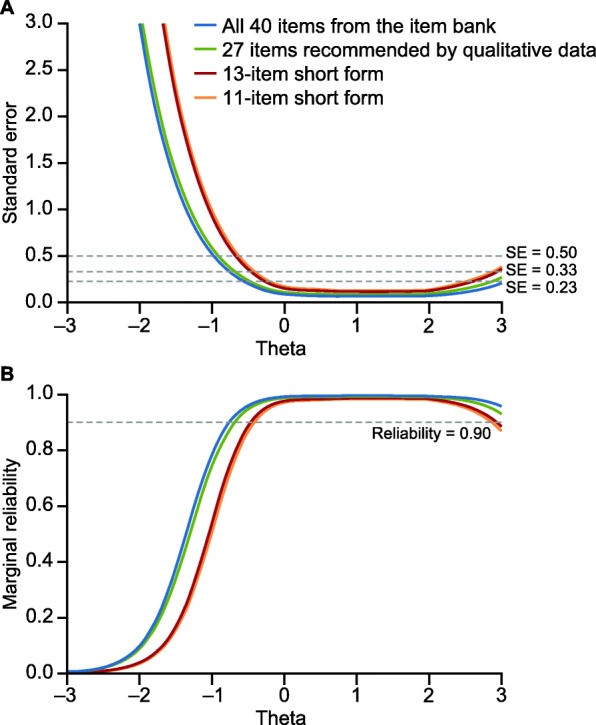
Standard error (**A**) and marginal reliability (**B**) of the 40-item PROMIS Pain Interference bank, the 27-item version recommended by the qualitative data, a 13-item candidate short form, and the final recommended 11-item short form. PROMIS, Patient-Reported Outcomes Measurement Information System; SE, standard error

To investigate whether the 13-item Pain Interference short form could be reduced further, additional analyses compared the latent variable IRT score distribution of the 13-item short form with candidate 12-item and 11-item versions in which one or two additional items had been removed. It was found that the combined removal of one item from the cognitive subdomain and one item from the fun/recreation/leisure subdomain would have only a minimal impact on the score distribution (range − 1.04 to 3.05) whilst retaining the performance properties of the 13-item short form. This 11-item short form had a standard error level of < 0.50 (equivalent to a marginal reliability > 0.80) throughout the range from − 0.6 to 3.0 (Fig. 2A and B) and a standard error level of < 0.33 (equivalent to a marginal reliability > 0.90) throughout the range from − 0.4 to 2.8 (Fig. 2A and B). Therefore, the 11-item Pain Interference short form is recommended for use in RA populations (Table 5).

**Table 5:** Recommended short forms of the pain interference (11 items) and sleep disturbance (7 items) item banks for use in a RA population

Item number	Item (rated on a 5-item scale from “not at all” to “very much”)
**PROMIS pain interference**	
PAININ9	How much did pain interfere with your day to day activities?
PAININ35	How much did pain interfere with your ability to make trips from home that kept you gone for more than 2 h?
PAININ29	How often was pain so severe you could not think of anything else?
PAININ20	How much did pain feel like a burden to you?
PAININ56	How irritable did you feel because of pain?
PAININ3	How much did pain interfere with your enjoyment of life?
PAININ47	How often did pain prevent you from standing for more than 30 min?
PAININ50	How often did pain prevent you from sitting for more than 30 min?
PAININ54	How often did pain keep you from getting into a standing position?
PAININ17	How much did pain interfere with your relationships with other people?
PAININ31	How much did pain interfere with your ability to participate in social activities?
**PROMIS sleep disturbance**	
SLEEP108	My sleep was restless
SLEEP115	I was satisfied with my sleep
SLEEP44	I had difficulty falling asleep
SLEEP71	I had trouble getting into a comfortable position to sleep
SLEEP110	I got enough sleep
SLEEP92	I woke up and had trouble falling back to sleep
SLEEP109	My sleep quality was … ^a^

*PROMIS* Patient-Reported Outcomes Measurement Information System

^a^Rated on a 5-item scale from “poor” to “very good”. PROMIS items© 2008–2021; reprinted with permission, David Cella

#### Sleep disturbance short form

**Fig. 3:**
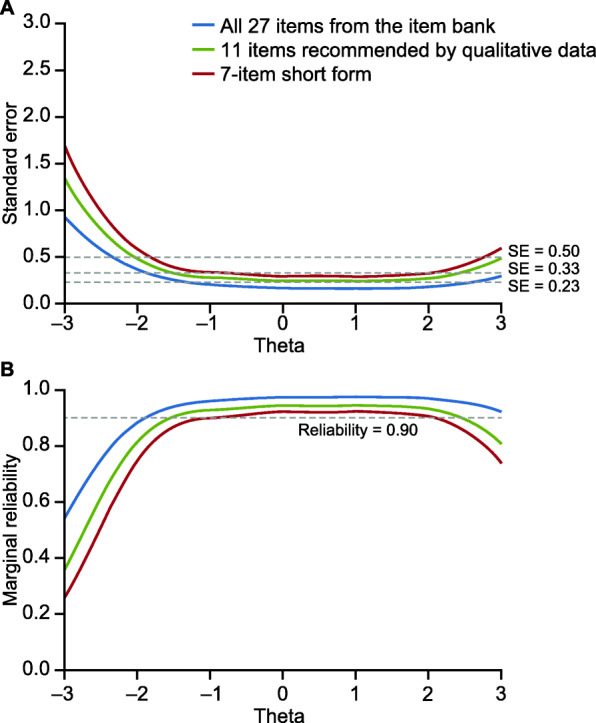
Standard error (**A**) and marginal reliability (**B**) of the 27-item PROMIS Sleep Disturbance bank, the 11-item version recommended by the qualitative data, and the final recommended 7-item short form. PROMIS, Patient-Reported Outcomes Measurement Information System; SE, standard error

Content evaluation of the 11 Sleep Disturbance items recommended from the qualitative interviews identified several items that were potentially redundant. For example, the items *‘My sleep was restful’* and *‘My sleep was restless’* were described by participants with RA as being redundant, and during psychometric evaluation they were deemed to be similar enough that the inclusion of both items in a short form was unnecessary. In this case, ‘*My sleep was restless’* was retained based on a comparison of their item threshold parameters on the range of the latent variable. This process resulted in 4 items being removed, providing a candidate Sleep Disturbance short form of 7 items. Psychometric evaluation found that this 7-item scale had good coverage over the range of the latent variable (range: − 2.01 to 2.82), and measurement precision was not overly compromised in comparison with the 11-item version. The 7-item short form had a standard error level of < 0.50 in the range from − 1.8 to 2.7 (equivalent to a marginal reliability of > 0.80) (Fig. 3A and B) and a standard error level of < 0.33 (equivalent to a marginal reliability > 0.90) through the range from − 1.1 to 2.0 (Fig. 3A and B).

Although the 7-item Sleep Disturbance short form was found to have good psychometric properties, an additional analysis was conducted to assess whether the addition of 1 item would result in an 8-item scale with notably improved performance. However, each of the 4 items removed from the initial 11-item scale only provided negligible improvements to the range of the latent variable in comparison with the 7-item short form. Consequently, the 7-item short form is recommended for use in RA populations (Table 5).

## Discussion

This study assessed the content validity of the PROMIS Pain Interference and Sleep Disturbance item banks in an RA population, and developed short forms consisting of a subset of items deemed most relevant to patients with RA, while also maintaining high measurement precision across a large range of the latent variable; the recommended short forms have the potential to be used in clinical studies of RA.

The development of the short forms in this study comprised qualitative cognitive debriefing to identify potential items, and quantitative psychometric evaluation of existing IRT item parameters to evaluate marginal reliability across the range of the latent variable and identify final versions of the short forms. While short forms for PROMIS Pain Interference and Sleep Disturbance have previously been developed [27, 36], they were not developed for use in the context of RA alone. The approach used in this study, including the selection of a particular item from a subset of conceptually redundant items using patient input, identified and validated the optimal items for use in clinical studies of RA treatments. These final items capture the concepts that are most important to the RA disease experience, and present them in a manner preferred by patients with RA. Furthermore, these refined short forms streamline the reporting process, and thus, reduce the respondent burden [37]. During concept elicitation, participants described several burdensome impacts of RA-related pain and sleep that affected their daily lives, including impacts to physical, social and emotional functioning. These findings provide novel insights into the complex relationship between sleep disturbances and RA symptoms, including pain, from the patient perspective. It was found that all patients described how their sleep was impacted by RA-related pain, such as making it difficult to find a comfortable sleeping position and experiencing a heightened awareness of the pain while lying in bed. For some participants, their sleep disturbance may have contributed to pain, such as inadvertent movements resulting in an onset of pain that caused them to awaken.

The cognitive debriefing interviews did not reveal any major problems with participants’ understanding of items, instructions, recall period, or response options in the full Pain Interference or Sleep Disturbance item banks, and there were no consistent or discernible patterns in missing concepts related to the instruments, indicating that PROMIS item banks provide a comprehensive selection of items to draw from. Participants reported that the majority of the items in the Pain Interference and Sleep Disturbance scales were relevant to their RA experience, although fewer items were considered not relevant in the Pain Interference bank (3/40 items) compared with the Sleep Disturbance bank (7/27 items).

By using cognitive debriefing to recommend items for psychometric evaluation, the initial set of items were those that participants had already indicated were clear, relevant, and unproblematic*.* Furthermore, a systematic approach to item reduction was applied to identify balanced Pain Interference and Sleep Disturbance short forms that have high relevance for RA and good psychometric properties. Importantly, the items selected for the Pain Interference and Sleep Disturbance short forms cover the areas of impact identified during the concept elicitation interviews. It has been noted that alternative PRO measures frequently used in RA studies may contain items that are not seen to be relevant to the disease by patients and physicians while other aspects, such as the impairment of work-related activities, are neglected [38–40]. The short forms developed in the present study used a combination of qualitative methods and psychometric evaluation to ensure that questions were relevant to patients with RA, comprehensible, and captured the scope of the full item bank with high reliability. A similar psychometric approach to item reduction has been used in the development of a PROMIS Depression short form [37].

Despite the strengths of the mixed-methods approach used, this study is not without limitations. Recruitment challenges with autoimmune diseases such as RA, which can involve extreme levels of fatigue and disease flares, resulted in some cancellations and a requirement to conduct a small number of interviews by phone (*n* = 5) to ensure that the desired sample size was achieved. However, this approach ensured that participants with severe RA and a range of clinical manifestations could be retained within the study population. Diagnosis and severity of RA was self-reported and not clinician-confirmed, but efforts were made during screening, recruitment and the interview process to mitigate any misclassification. Participants were requested to bring their RA medication to the interviews, which indirectly confirmed the diagnosis and allowed disease severity to be inferred: 56% of participants were receiving biologic-containing therapies, which are often prescribed for patients with moderate or severe RA [41], whereas 41% were receiving csDMARDs only. Another limitation is that only patients who were fluent in English from the United States were included, with most participants located in the Northeastern and Midwestern regions. Participants were mostly located near metropolitan areas to allow them to travel to the qualitative research facilities and to ensure that an adequate sample size could be recruited for in-person interviews. A potential limitation of the psychometric evaluation was that candidate items were evaluated using item parameters that were estimated by PROMIS investigators and calibrated in a general population [25]. Our analyses of measurement precision assume that the PROMIS item parameters also pertain to patients with RA. Collection of PROMIS data from patients with RA and tests of differential item functioning would provide the necessary information to understand whether the items perform similarly in an RA population as they do in the more general population.

## Conclusions

Findings from this study provide valuable information from a patient perspective about impacts of pain and sleep disturbances associated with RA, and support the use of the PROMIS Pain Interference and Sleep Disturbance item banks in RA. Short-form versions of the item banks were identified for use in RA populations, and have the potential to be utilized in clinical studies. A rigorous approach to item selection was used to ensure that all items in the short forms were relevant to patients with RA, whilst also maximizing measurement precision across the latent variable range. This approach may allow for the detection of more nuanced aspects of treatment benefit. These short forms are being used in the Phase 3 clinical development program for otilimab [42], a high-affinity recombinant human monoclonal antibody that binds to and inhibits human granulocyte-macrophage colony-stimulating factor [43, 44].

## Supplementary Information

**Additional file 1 : Supplementary Table 1.** Items in the PROMIS Pain Interference bank reported as redundant and, if applicable, the items reported to be the most preferred. **Supplementary Table 2.** Items in the PROMIS Sleep Disturbance bank reported as redundant and, if applicable, the items reported to be the most preferred.

## Data Availability

Information on GlaxoSmithKline’s (GSK’s) data sharing commitments and requesting access to anonymized individual participant data and associated documents from GSK-sponsored studies can be found at www.clinicalstudydatarequest.com.

## Abbreviations

DMARD: Disease-modifying anti-rheumatic drug
IRT: Item response theory
ISPOR: International Society for Pharmacoeconomics and Outcomes Research
NRS: Numeric rating scale
PRO: Patient-reported outcome
PROMIS: Patient-Reported Outcomes Measurement Information System
RA: Rheumatoid arthritis
SD: Standard deviation
SE: Standard error
VAS: Visual analogue scale
