# Supplementary material for: Qualitative and psychometric approaches to evaluate the PROMIS pain interference and sleep disturbance item banks for use in patients with rheumatoid arthritis
Source: J Patient Rep Outcomes. 2021 Jul 6;5:52. doi: 10.1186/s41687-021-00318-w (PMC8260648; doi:10.1186/s41687-021-00318-w)
Supplement: Supplementary file 1 — Additional file 1 : Supplementary Table 1. Items in the PROMIS Pain Interference bank reported as redundant and, if applicable, the items reported to be the most preferred. Supplementary Table 2. Items in the PROMIS Sleep Disturbance bank reported as redundant and, if applicable, the items reported to be the most preferred. [file 41687_2021_318_MOESM1_ESM.docx]

# Supplementary data

**Supplementary Table 1: Items in the PROMIS Pain Interference bank reported as redundant and, if applicable, the items reported to be the most preferred**

| **PROMIS Pain Interference Item** | **Redundant** | **Preferred** | **Comment** |
| --- | --- | --- | --- |
| PAININ34: How much did pain interfere with your household chores? | 19 | 5 | Five participants preferred PAININ34 in comparison with PAININ22 and PAININ48. |
| PAININ48: How much did pain interfere with your ability to do household chores? | 17 | 9 | Nine participants preferred this item’s wording “ability to do” household chores in comparison with PAININ22 and PAININ34. |
| PAININ22: How much did pain interfere with work around the home? | 17 | 6 | Six participants preferred SLEEP22 over SLEEP34 and SLEEP48; five reported this item the same as household chores, while one reported this item included chores and other activities such as gardening. |
| PAININ31: How much did pain interfere with your ability to participate in social activities? | 16 | 5 | Five preferred PAININ31 “ability to participate” over PAININ36 “enjoyment |
| PAININ46: How often did pain make it difficult for you to plan social activities? | 14 | 3 | Three preferred this item over PAININ52. |
| PAININ10: How much did pain interfere with your enjoyment of recreational activities? | 13 | 5 | Five participants preferred PAININ10; three preferred to keep PAININ5 and PAININ10 as separate items, though two preferred to eliminate PAININ5 and PAININ12 because PAININ10 was more relatable. |
| PAININ52: How often was it hard to plan social activities because you didn't know if you would be in pain? | 12 | 5 | Five preferred this item over PAININ46. |
| PAININ36: How much did pain interfere with your enjoyment of social activities? | 12 | 4 | Four preferred this item; three preferred this item over PAININ31 and one preferred this item over PAININ38. |
| PAININ12: How much did pain interfere with the things you usually do for fun? | 11 | 2 | Two participants preferred PAININ12 in comparison with PAININ5, PAININ10, and PAININ31 as they reported it covered leisure, recreational, and social activities and it was simple wording to understand. |
| PAININ26: How often did pain keep you from socializing with others? | 10 | 4 | Four preferred this item; two preferred this item over PAININ38, one preferred this item over PAININ5 (leisure activities), and one preferred this item over PAININ31 and PAININ36. |
| PAININ18: How much did pain interfere with your ability to work (include work at home)? | 9 | 6 | Nine participants identified this as redundant but were focused on “work at home” due to retirement. Six preferred this item in comparison with PAININ22, PAININ34, and PAININ48. |
| PAININ38: How often did you avoid social activities because it might make you hurt more? | 9 | 2 | Two preferred this item; one preferred it over PAININ53 and one preferred it over all social activities items. |
| PAININ5: How much did pain interfere with your ability to participate in leisure activities? | 8 | 6 | Six participants preferred PAININ5; three preferred to keep PAININ5 and PAININ10 over PAININ12. Two preferred PAININ5 over PAININ10 because ‘leisure activities’ was more relatable to them on a daily basis. One participant preferred PAININ5 over PANIN31 and one preferred PAININ5 over PAININ12. |
| PAININ17: How much did pain interfere with your relationships with other people? | 7 | 3 | Three participants preferred PAININ17 because it was more general and encompassed PAININ13. |
| PAININ53: How often did pain restrict your social life to your home? | 7 | 1 | One reported this item should be retained because it seemed different from the other social functioning items. |
| PAININ8: How much did pain interfere with your ability to concentrate? | 5 | 5 |  |
| PAININ24: How often was pain distressing to you? | 5 | 2 | Five participants reported PAININ24 as redundant; two reported redundant with PAININ16 and PAININ32, one reported PAININ8, PAININ37, and interference with social activities covered whether pain was distressing, one reported redundant with PAININ56, one reported redundant with PAININ20. Two participants preferred this item over PAININ56 and PAININ20. |
| PAININ50: How often did pain prevent you from sitting for more than 30 minutes? | 5 | 2 | Five participants reported this item was redundant; three reported redundant with PAININ36 and PAININ39 and two reported redundant with just PAININ36. Two participants preferred this item. |
| PAININ51: How often did pain prevent you from sitting for more than 10 minutes? | 5 | 2 |  |
| PAININ1: How difficult was it for you to take in new information because of pain? | 5 | 0 |  |
| PAININ13: How much did pain interfere with your family life? | 5 | 0 |  |
| PAININ14: How much did pain interfere with doing your tasks away from home (e.g., getting groceries, running errands)? | 4 | 2 |  |
| PAININ47: How often did pain prevent you from standing for more than 30 minutes? | 4 | 2 |  |
| PAININ42: How often did pain prevent you from standing for more than one hour? | 4 | 1 |  |
| PAININ32: How often did pain make you feel discouraged? | 3 | 2 | Two participants preferred PAININ32 over PAININ16. |
| PAININ3: How much did pain interfere with your enjoyment of life? | 3 | 1 |  |
| PAININ16: How often did pain make you feel depressed? | 3 | 1 | One participant preferred PAININ16 over PAININ24. |
| PAININ55: How often did pain prevent you from sitting for more than one hour? | 3 | 1 | One participant preferred this item and PAININ36 over PAININ35. |
| PAININ35: How much did pain interfere with your ability to make trips from home that kept you gone for more than 2 hours? | 3 | 0 |  |
| PAININ49: How much did pain interfere with your ability to remember things? | 3 | 0 |  |
| PAININ6: How much did pain interfere with your close personal relationships? | 2 | 2 |  |
| PAININ37: How often did pain make you feel anxious? | 2 | 1 |  |
| PAININ9: How much did pain interfere with your day to day activities? | 2 | 0 |  |
| PAININ56: How irritable did you feel because of pain? | 1 | 1 |  |
| PAININ20: How much did pain feel like a burden to you? | 1 | 0 |  |
| PAININ54: How often did pain keep you from getting into a standing position? | 1 | 0 |  |
| PAININ11: How often did you feel emotionally tense because of your pain? | 0 | 0 |  |
| PAININ19: How much did pain make it difficult to fall asleep? | 0 | 0 |  |
| PAININ29: How often was your pain so severe you could think of nothing else? | 0 | 0 |  |
| PAININ40: How often did pain prevent you from walking more than 1 mile? | 0 | 0 |  |

PROMIS items © 2008-2021; reprinted with permission, David Cella.

**Supplementary Table 2: Items in the PROMIS Sleep Disturbance bank reported as redundant and, if applicable, the items reported to be the most preferred**

| **PROMIS Sleep Disturbance Item** | **Redundant** | **Preferred** | **Comment** |
| --- | --- | --- | --- |
| SLEEP87: I had trouble staying asleep | 7 | 1 | Mostly reported as redundant with SLEEP 92. SLEEP92 was most often preferred as it is more specific and provides more detail |
| SLEEP92: I woke up and had trouble falling back to sleep | 6 | 3 | Of those that indicated a preference (n=4), most (n=3) indicated that they preferred this item over SLEEP50 and SLEEP87. |
| SLEEP108: My sleep was restless | 5 | 4 | Four participants preferred item SLEEP108 versus SLEEP107, SLEEP86, and SLEEP105. |
| SLEEP105: My sleep was restful | 5 | 3 | Three participants preferred SLEEP105 versus SLEEP116 and SLEEP108. |
| SLEEP90: I had trouble sleeping | 5 | 1 | One participant reported preferring SLEEP90 in comparison to SLEEP87. Some were unsure whether this meant falling asleep, staying asleep or just sleeping and five felt that it was redundant with the other three questions (SLEEP50, SLEEP87, and SLEEP92) as answering yes to either would indicate that someone had trouble sleeping. |
| SLEEP116: My sleep was refreshing | 4 | 2 | Two participants preferred SLEEP116 but one of the two participants was comparing this to “my sleep is satisfied” rather than restful. |
| SLEEP86: I tossed and turned at night | 4 | 1 | One participant preferred SLEEP86 over SLEEP108 because it was more specific. |
| SLEEP107: My sleep was deep | 4 | 0 |  |
| SLEEP68: I felt worried at bedtime | 4 | 0 |  |
| SLEEP50: I woke up too early and could not fall back asleep | 4 | 0 | No one preferred SLEEP50. One indicated some confusion with this item, as they felt that “early” differed for everyone. |
| SLEEP106: My sleep was light | 3 | 0 | No participant preferred SLEEP106. One indicated that they were a light sleeper naturally, regardless of their RA pain. |
| SLEEP115: I was satisfied with my sleep | 2 | 1 |  |
| SLEEP67: I worried about not being able to fall asleep | 2 | 1 |  |
| SLEEP69: I had trouble stopping my thoughts at bedtime | 2 | 0 |  |
| SLEEP42: It was easy for me to fall asleep | 1 | 1 |  |
| SLEEP125: I felt lousy when I woke up | 1 | 0 |  |
| SLEEP20: I had a problem with my sleep | 1 | 0 |  |
| SLEEP71: I had trouble getting into a comfortable position to sleep | 1 | 0 |  |
| SLEEP110: I got enough sleep | 1 | 0 |  |
| SLEEP45: I laid in bed for hours waiting to fall asleep | 1 | 0 |  |
| SLEEP44: I had difficulty falling asleep | 0 | 0 |  |
| SLEEP65: I felt physically tense at bedtime | 0 | 0 |  |
| SLEEP70: I felt sad at bedtime | 0 | 0 |  |
| SLEEP72: I tried hard to get to sleep | 0 | 0 |  |
| SLEEP78: Stress disturbed my sleep | 0 | 0 |  |
| SLEEP93: I was afraid I would not get back to sleep after waking up | 0 | 0 |  |
| SLEEP109: My sleep quality was | 0 | 0 |  |

PROMIS items © 2008-2021; reprinted with permission, David Cella.
